# Tuberculosis screening in patients with HIV: use of audit and feedback to improve quality of care in Ghana

**DOI:** 10.3402/gha.v9.32390

**Published:** 2016-08-26

**Authors:** Stephanie Bjerrum, Frank Bonsu, Nii Nortey Hanson-Nortey, Ernest Kenu, Isik Somuncu Johansen, Aase Bengaard Andersen, Lars Bjerrum, Dorte Jarbøl, Anders Munck

**Affiliations:** 1 Department of Infectious Diseases, Odense University Hospital, Odense, Denmark; 2 Institute of Clinical Research, Faculty of Health Sciences, University of Southern Denmark, Odense, Denmark; 3 National Tuberculosis Control Programme, Disease Control and Prevention Department, Ghana Health Services, Korle-Bu Teaching Hospital, Accra, Ghana; 4 Department of Medicine–Fevers Unit, Korle-Bu Teaching Hospital, Accra, Ghana; 5 School of Public Health, University of Ghana, Legon, Accra, Ghana; 6 Department of Infectious Diseases, Copenhagen University Hospital, Rigshospitalet, Copenhagen, Denmark; 7 Department of Public Health, University of Copenhagen, Copenhagen, Denmark; 8 Department of Public Health, University of Southern Denmark, Odense, Denmark

**Keywords:** HIV, tuberculosis, screening, quality of healthcare, clinical practice, audit, feedback

## Abstract

**Background:**

Tuberculosis screening of people living with HIV (PLHIV) can contribute to early tuberculosis diagnosis and improved patient outcomes. Evidence-based guidelines for tuberculosis screening are available, but literature assessing their implementation and the quality of clinical practice is scarce.

**Objectives:**

To assess tuberculosis screening practices and the effectiveness of audit and performance feedback to improve quality of tuberculosis screening at HIV care clinics in Ghana.

**Design:**

Healthcare providers at 10 large HIV care clinics prospectively registered patient consultations during May and October 2014, before and after a performance feedback intervention in August 2014. The outcomes of interest were overall tuberculosis suspicion rate during consultations and provider adherence to the International Standards for Tuberculosis Care and the World Health Organizations’ guidelines for symptom-based tuberculosis screening among PLHIV.

**Results:**

Twenty-one healthcare providers registered a total of 2,666 consultations; 1,368 consultations before and 1,298 consultations after the feedback intervention. Tuberculosis suspicion rate during consultation increased from 12.6 to 20.9% after feedback (odds ratio, OR 1.83; 95% confidence interval, CI: 1.09–3.09). Before feedback, sputum smear microscopy was requested for 58.7% of patients with suspected tuberculosis, for 47.2% of patients with cough ≥2 weeks, and for 27.5% of patients with a positive World Health Organization (WHO) symptom screen (any of current cough, fever, weight loss or night sweats). After feedback, patients with a positive WHO symptom screen were more likely to be suspected of tuberculosis (OR 2.21; 95% CI: 1.19–4.09) and referred for microscopy (OR 2.71; 95% CI: 1.25–5.86).

**Conclusions:**

A simple prospective audit tool identified flaws in clinical practices for tuberculosis screening of PLHIV. There was no systematic identification of people with suspected active tuberculosis. We found low initial tuberculosis suspicion rate compounded by low referral rates of relevant patients for sputum smear microscopy. Adherence to recommended standards and guidelines for tuberculosis screening improved after performance feedback.

## Introduction

Tuberculosis remains a leading cause of morbidity and mortality among people living with HIV (PLHIV) (1). A review of post-mortem studies among HIV-infected individuals showed that tuberculosis was implicated in 33% of all HIV-associated adult deaths in sub-Saharan Africa, with almost half of the fatal cases undiagnosed in life (2). In Ghana, the HIV epidemic is moderate with a prevalence of 1.4% (3). The overall tuberculosis prevalence is 282/100,000 (4), and low tuberculosis case detection rate overall and among PLHIV has been identified as main challenges in the country (5).

To increase tuberculosis case detection among PLHIV in resource-constrained areas, great effort has been made to evaluate tuberculosis screening algorithms and diagnostic tests (6–8). In 2011, the World Health Organization (WHO) endorsed a four-symptom-based clinical algorithm as part of their guidelines for tuberculosis screening of PLHIV (9). Moreover, The International Standards for Tuberculosis Care (ISTC) published by WHO and the American Thoracic Society is available to promote high-quality services (10, 11). However, there is little information on the quality of tuberculosis screening practices in the context of routine care. To date, studies have focused on coverage of tuberculosis screening based on retrospective record reviews or routine health information (12–15). These studies reported great variation in coverage with a general increase over time and after education of healthcare providers. Low referral rates for tuberculosis diagnostic test were reported in some of the studies (13, 15). Still, the need remains to assess healthcare providers’ initial tuberculosis suspicion and decision to refer patients for further diagnostic tests as well as identify strategies to improve quality of care under real-life conditions.

Clinical audit and feedback is a tool to assess clinical practice and improve the quality of care, and has been found effective to close the gap between routine practice and recommended guidelines (16, 17). Audit has previously been used to assess the quality of diagnosis of smear-negative pulmonary tuberculosis (18) and to improve general tuberculosis diagnostic services in Latin America (19). In collaboration with the National Tuberculosis Control Programme (NTP) in Ghana, we conducted a quality improvement study at 10 large HIV care clinics. The aim was to assess healthcare providers’ adherence to widely recommended standards for HIV-associated tuberculosis screening and evaluate if audit and feedback could improve performance. We used a simple tool for audit and feedback based on prospective self-registration of healthcare providers’ clinical practice focusing on tuberculosis suspicion and referral for sputum smear microscopy. We use the Standards for Quality Improvement Reporting Excellence (SQUIRE 2.0) to report our results (20).

## Methods

### Design

This is a prospective study with a one-group pre- and post-evaluation of audit and feedback to address and improve the quality of tuberculosis screening practices at HIV care clinics.

### Study sites and context

The study was conducted at 10 large HIV care clinics located in major hospitals from three regions in the southern zone of Ghana. The clinics were selected from the national list of public HIV care clinics based on the following criteria: >3,000 PLHIV enrolled at the clinics; access to X-ray and sputum smear microscopy for acid-fast bacilli at the hospital premises; and availability of a tuberculosis care clinic or a clinic with integrated delivery of HIV and tuberculosis services at the hospital premises. Two of the HIV care clinics were affiliated to a major teaching hospital. Half of the study sites had access to a specific clinic with integrated HIV/tuberculosis services at the hospital.

In Ghana, the NTP and the National AIDS Control Programme (NACP) implement HIV and tuberculosis collaborative services and perform routine monitoring and support to health facilities. As in many resource-constrained areas, sputum smear microscopy is the most widely used tuberculosis diagnostic test, while new diagnostic test like the Xpert MTB-RIF is being scaled up (21). National guidelines recommend regular symptom-based tuberculosis screening of PLHIV including questions on cough and duration (≥2 weeks or <2 weeks), cough with blood, weight loss, night sweats, fever, chest pain, and history of any contact to persons known with tuberculosis (22, 23). All individuals with cough ≥2 weeks should be referred for sputum smear microscopy for acid-fast bacilli. The WHO guidelines available for tuberculosis screening in PLHIV recommend that individuals reporting any weight loss, fever, night sweats, and current cough (of any duration) should be referred for further tuberculosis investigations (9). According to the WHO guidelines, isoniazid preventive treatment (IPT) should be offered to those individuals reporting none of these symptoms, but IPT is not yet implemented in Ghana.

At the time the study was conducted, 4 of 10 HIV care clinics had access to Xpert MTB/RIF, but none used Xpert MTB/RIF as part of routine diagnostic services for tuberculosis.

### Participants

We invited all healthcare providers involved in tuberculosis screening at the 10 HIV care clinics to participate in the study. Medical doctors and nurses, but also physician assistants and disease control officers, were invited for participation if active in tuberculosis screening. Participants were asked to register consultations with PLHIV during May and October 2014.

### Data collection – Audit Project Odense

We used the Audit Project Odense (APO) method for audit and compilation of feedback. The method is originally developed for quality improvement in general practice but has since been used widely (24–27). The APO method is based on prospective self-registration of clinical practice during patient consultations using a simple paper form. The form used for this study (Supplementary material A) was designed for registrations to take <2 min per consultation. The healthcare providers were asked to register the first 15 consultations with PLHIV daily for 2–4 weeks before and after a feedback intervention – in the following referred to as the first and second audit, respectively. Data registered during consultation included demographic characteristics of patients (age and sex); the consultation type (initial assessment of a newly diagnosed HIV-infected individual vs. follow-up consultations); antiretroviral treatment (ART) status of patients (currently receiving ART, defaulted ART for more than 1 month since last consultation, or never received ART); tuberculosis-related signs and symptoms presented during consultation (cough, weight loss, night sweats, fever); the healthcare providers’ main suspicion or diagnosis (including tuberculosis, pneumonia, upper respiratory tract infection); and prescribed investigations. Healthcare providers were carefully instructed orally and in writing (Supplementary material B) on how to fill in the registration form before the first audit and received a follow-up visit by the research team during registration. They were further asked to complete a form with background information about themselves and the HIV care clinic they represented.

### Feedback

**Fig. 1:**
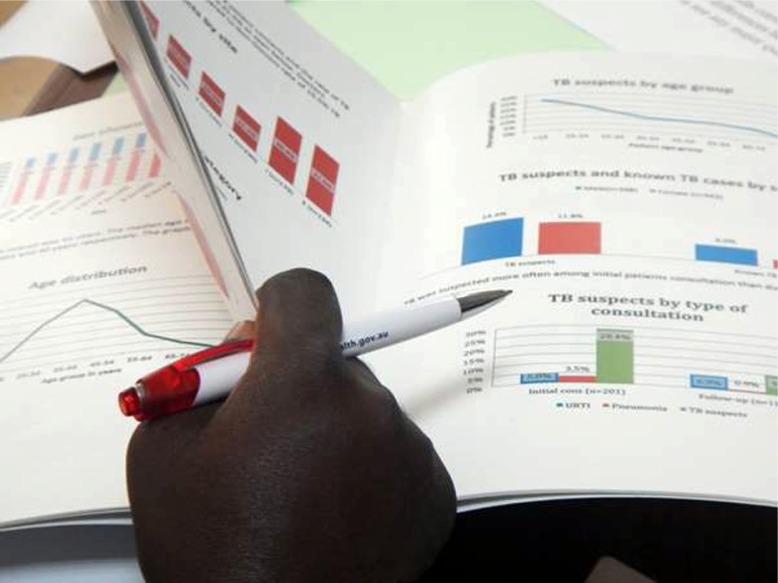
Feedback report.

Based on the results of the first audit, performance feedback was developed for each of the healthcare providers. This included a feedback report where the results from the first audit were summarized in words, tables, and diagrams focusing on the healthcare providers’ overall practice for suspecting tuberculosis and referral of tuberculosis suspects for further investigations (Fig. 1). Moreover, a personal feedback sheet was developed for each participant to enable the individual participant to compare their own results with those of their peers. The feedback report and personal feedback sheet were distributed to the healthcare providers prior to a one-day feedback workshop held in August 2015. Here, the feedback report was presented and discussed. The findings were put in context with preliminary results from the national tuberculosis prevalence survey, relevant studies on the area, and the available guidelines for tuberculosis screening. The workshop further comprised group work sessions to identify challenges for adherence to guidelines and possible areas for quality improvements. The principal investigator (SB), a consultant for the APO method (LB), and key persons from the NTP (FB and NNHN) and NACP facilitated the feedback workshop.

### Study outcome measures and definitions

The primary outcome measure was to determine and characterize healthcare providers’ tuberculosis suspicion rate. We defined a series of secondary outcome measures based on recommended standards for tuberculosis screening of PLHIV and adherence rates to these (Box 1). The performance standards were selected in collaboration with key informants at NTP and NACP and were based on the ISTC and WHO guidelines (9, 10). A positive WHO tuberculosis symptom screen (‘WHO-TB’) is defined as PLHIV presenting with any of the following four symptoms: fever, cough (of any duration, i.e. both the variable cough <2 weeks and cough ≥2 weeks), night sweat, or weight loss (9).

Box 1 **Outcome measures compared to recommendations**

**Table:** 

**Primary outcome measure**	
Tuberculosis suspicion rate	
**Secondary outcome measures**	**Recommended standards**

Proportion of individuals with cough ≥2 weeks suspected of tuberculosis	ISTC, Standard 1: All persons with otherwise unexplained cough lasting 2–3 weeks or more should be evaluated for tuberculosis^a^
Proportion of individuals with cough ≥2 weeks referred for sputum smear microscopy Proportion of individuals who are suspected of tuberculosis referred for sputum smear microscopy	ISTC, Standard 2: All patients (adults, adolescents, and children who are capable of producing sputum) suspected of having pulmonary tuberculosis should have at least two, and preferably three, sputum specimens obtained for microscopic examination^a^
Proportion of individuals with a positive WHO-TB screen suspected of TB Proportion of individuals with a positive WHO-TB screen referred for sputum smear microscopy	WHO: Adults and adolescents living with HIV and screened for TB with a clinical algorithm and who report any one of the symptoms of current cough, fever, weight loss, or night sweats may have active TB and should be evaluated for TB and other diseases^b^¶

a International Standards for Tuberculosis Care, 2006 (10).

b WHO guidelines for tuberculosis screening among PLHIV, 2011 (9).

### Statistical analysis

Descriptive analysis was used to characterize the participating healthcare providers and the study population using chi-square test for categorical variables and Wilcoxon rank-sum for continuous variables. We used logistic regression models with robust standards errors (SE) to obtain point estimates for the outcome measures with 95% confidence intervals (CI) pre- and post-feedback. As feedback targeted the healthcare providers and not the HIV care clinic, we adjusted for clustering of registrations by the same provider. Difference in outcome measures was analysed for statistical significance using logistic regression with results from the first audit used as reference. Statistical significance was defined as a two-sided *p*-value less than 0.05, and all analyses were conducted using the statistical software package STATA™ (version 13.1).

### Ethical clearance

The project was approved by the Ghana Health Service Ethical Review Committee (GHS-ERC: 09/03/14), the Ethical and Protocol Review Committee, University of Ghana Medical School (MS-Et./M.4–P 3.3/2012-13) and evaluated by the Developing Country Committee of the Danish National Committee on Health Research Ethics (No. 1302133/1206169). The healthcare providers signed a written informed consent form before participation. We did not register any patient identifiable information apart from age and sex nor expose patients to any intervention, and hence, there were no requirements to collect informed consent from patients contributing data.

## Results

### Healthcare providers’ details

Healthcare providers (*n*=22) from 10 HIV care clinics participated in the study including 10 medical doctors, eight nurses, three physician assistants, and one disease control officer. Registrations from one physician assistant were excluded from analysis, since the person failed to register according to instructions. The healthcare providers reported a medium of 5 years (IQR 2–10) of expertise in a HIV care clinic and had a median of 40 (30–70) outpatient consultations per day. All providers received feedback and participated in the feedback workshop; 16 providers participated in both audits.

### Consultations

Data from 2,666 consultations with PLHIV were included in the study based on registration of 1,368 consultations in the first audit and 1,298 consultations in the second audit after performance feedback (Table 1). Overall, consultations comprised of 2,185 (81.9%) follow-up visits and 439 (16.5%) initial assessments of new HIV-positive patients. The majority of patients consulted were females 1,910 (71.6%) and most were receiving ART 1,869 (70.1%). Baseline variables differed between the two audits with regard to consultation type, sex of the individuals, proportion of patients that had defaulted ART, and patients presenting with weight loss or cough <2 weeks. After adjusting for clustering to healthcare provider, only the sex of the patients remained significantly different between the audits.

**Table 1:** Consultation and patient characteristics at first and second audit

	Overall (*N*=2,666)	First audit (*N*=1,368)		Second audit (*N*=1,298)	
			
	*n* (%)	*n*	%	*n*	%
Consultation					
Initial	439 (16.5)	201	14.7	238	18.3
Follow-up	2,185 (81.9)	1,140	83.3	1,045	80.5
Unknown	42 (1.6)	27	2.0	15	1.2
Sex					
Male	741 (27.8)	418	30.6	323	24.9
Female	1,910 (71.6)	943	68.9	967	74.5
Unknown	15 (0.6)	7	0.5	8	0.6
Age in years					
Median (IQR)	40 (33–48)	41 (33–48)		40 (33–48)	
HIV Treatment status					
Receiving ART	1,869 (70.1)	940	68.7	929	71.6
ART naive	580 (21.8)	291	21.3	289	22.2
Defaulted ART (>1 month)	78 (2.9)	51	3.7	27	2.1
Unknown	139 (5.2)	86	6.3	53	4.1
Signs and symptoms					
Weight loss	374 (14.0)	167	12.2	207	16.0
Fever	397 (14.9)	199	14.6	198	15.3
Cough <2 weeks	337 (12.6)	146	10.7	191	14.7
Cough ≥2 weeks	298 (11.2)	144	10.5	154	11.9
Night sweats	172 (6.5)	80	5.9	92	7.1
Positive WHO-TB screen**a**	908 (34.1)	444	32.5	464	35.8

ART, antiretroviral therapy.

a Positive WHO symptoms screen if presence of any of the following symptoms: current cough, fever, weight loss, or night sweat (9).

### Tuberculosis suspicion rate

In the first audit, tuberculosis was suspected in 172/1,368 (12.6%) consultations with rates varying from 0.5–35.7% across the healthcare providers. The tuberculosis suspicion rate was higher in consultations with new HIV-positive patients than at follow-up (28.4% vs. 9.9%, odds ratio, OR 3.60; 95% CI: 2.33–5.55). Moreover, patients not receiving ART and patients who had defaulted ART for more than 1 month were more likely to be suspected of tuberculosis than patients receiving ART. Patients aged above 55 years were less likely to be suspected for tuberculosis. The tuberculosis suspicion rate appeared substantially lower for doctor-led consultations than for consultations led by the other staff categories combined, but the difference was borderline significant when accounting for clustering (15.5% vs. 9.5% OR 1.75; 95% CI: 0.99–3.11) (Table 2).

**Table 2:** Tuberculosis suspicion rate overall and by subgroups shown for the first and second audit

	First audit						Second audit					
		
Proportion of TB suspects	*n*/*N*	%	OR^a^	95% CI		*P*	*n*/*N*	%	OR^a^	95% CI		*P*
Overall	172/1,368	12.6					271/1,298	20.9%				
By staff category												
Medical doctors	63/665	9.5	ref				65/411	15.8%	ref			
Non-doctors	109/703	15.5	1.75	0.99	3.11	0.054	206/887	23.2%	1.61	0.54	4.79	0.391
By consultation												
Follow-up	113/1,140	9.9	ref				140/1,045	13.4%	ref			
Initial	57/201	28.4	3.60	2.33	5.55	**<0.001**	128/238	53.8%	7.52	3.94	14.34	**<0.001**
By patient sex												
Male	60/418	14.4	ref				84/323	26.0%	ref			
Female	111/943	11.8	0.80	0.54	1.18	0.251	186/967	19.2%	0.68	0.52	0.88	**0.004**
By age (years)												
0–17	10/45	22.2	1.51	0.56	4.06	0.416	5/25	20.0%	0.66	0.23	1.87	0.433
18–34.9	57/358	15.9	ref				92/335	27.5%	ref			
35–54.9	87/794	11.0	0.65	0.39	1.07	0.093	146/768	19.0%	0.62	0.39	0.99	**0.044**
≥55	17/170	10.0	0.59	0.38	0.92	**0.020**	27/167	16.2%	0.51	0.25	1.06	0.069
By ART status												
Receiving ART	87/940	9.3	ref				113/929	12.2%	ref			
ART naive	58/291	19.9	2.44	1.47	4.05	**0.001**	123/289	42.6%	5.35	3.09	9.27	**<0.001**
Defaulted ART (>1 month)	14/51	27.5	3.71	1.72	8.02	**0.001**	15/27	55.6%	9.03	3.14	25.91	**<0.001**

ART, antiretroviral treatment.

a OR: Odds ratio adjusted for clustering of registrations within health provider.

Non-doctors includes nurses (*n*=8), physician assistants (*n*=2), and disease control officer (*n*=1).

Missing values excluded from analysis; sex (*n*=15), age (*n*=4), consultation (*n*=42), ART status (*n*=139).

*P*-values in bold indicate values <0.05.

In the second audit, tuberculosis was suspected in 271/1,298 (20.9%) consultations. Tuberculosis suspicion rates were higher in consultation with patients of male sex, patients newly diagnosed with HIV, patients not receiving ART, or patients who defaulted ART. Patients in the age groups 35–55 years and above were less likely to be suspected of tuberculosis than patients aged 18–34 years, although borderline significant for the age group above 55 years (Table 2).

**Fig. 2:**
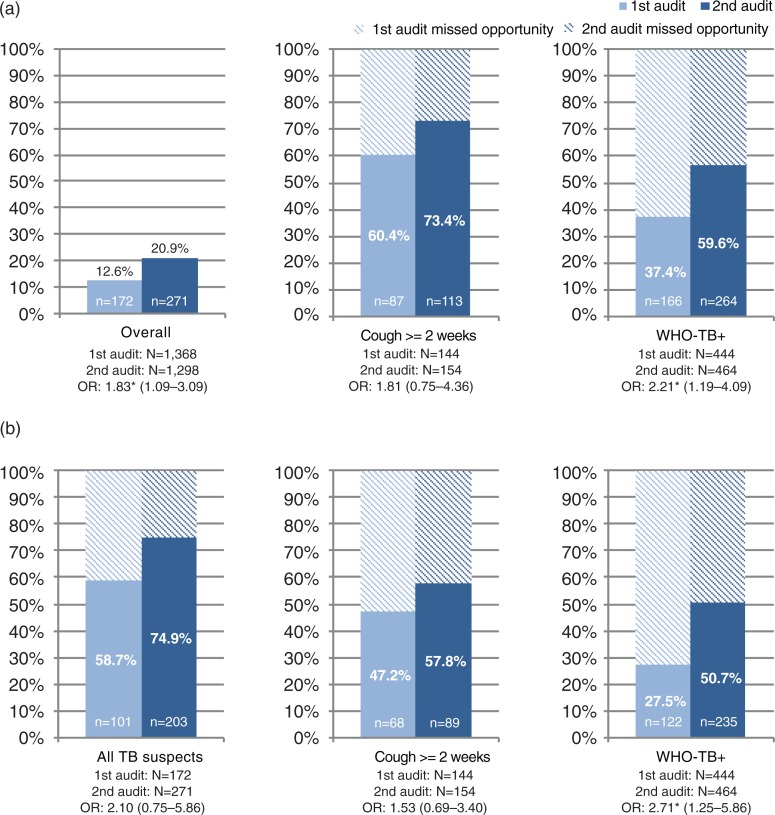
Tuberculosis suspicion rate and adherence to standards for tuberculosis screening and referral for sputum smear microscopy. (a) Tuberculosis suspicion rate among healthcare providers at first audit and second audit. (b) Referral rate for sputum smear microscopy among healthcare providers at first audit and second audit. Percentages are given as n/N. OR: Odds ratio shown with results from audit 1 as reference, adjusted for clustering of registrations within the health provider. WHO-TB+: Positive WHO symptoms screen defined as presence of any of the following symptoms; current cough, fever, weight loss or night sweats (9). Missing values excluded from analysis; sex (*n*=15), age (*n*=4), consultation (*n*=42), ART status (*n*=139).

The increase in tuberculosis suspicion rate from 12.6 to 20.9% after feedback was significant (OR 1.83; 95% CI: 1.09–3.09) (Fig. 2).

### Adherence to standards for tuberculosis screening and referral for sputum smear microscopy

The first audit highlighted wide gaps between practices of tuberculosis screening and applicable performance standards. The healthcare providers suspected tuberculosis in 87/144 (60.4%) patients presenting with cough ≥2 weeks and in 166/444 (37.4%) patients with a positive WHO-TB screen. Sputum smear microscopy was requested for 101/172 (58.7%) patients suspected of tuberculosis, for 68/144 (47.2%) patients presenting with cough ≥2 weeks, and for 122/444 (27.5%) patients with a positive WHO-TB screen (Fig. 2).

In the second audit, providers’ tuberculosis suspicion rate increased from 60.4 to 73.4% in patients presenting with cough ≥2 weeks and from 37.4 to 56.9% in patients with a positive WHO-TB screen. The increase in tuberculosis suspicion rate was statistically significant for patients with a positive WHO-TB screen (OR 2.21; 95% CI: 1.19–4.09), but not for those with cough ≥2 weeks (OR 1.81; 95% CI: 0.75–4.36). Referral for sputum smear microscopy increased to 74.9% in patients suspected of tuberculosis, to 57.8% in patients presenting with cough ≥2 weeks, and to 50.7% in patients with a positive WHO-TB screen. The increase was significant in referral of patients with a positive WHO-TB screen (OR 2.71; 95% CI: 1.25–5.86), but not for the other groups.

## Discussion

In this study, a simple tool for audit and feedback was integrated into the daily work of healthcare providers at 10 large HIV care clinics in Ghana. The first audit identified low tuberculosis suspicion rates and a marked gap between current practices and widely recommended standards for tuberculosis screening. After feedback to healthcare providers, we observed increased tuberculosis suspicion rates and improved the quality of tuberculosis screening performance. To our knowledge, this is the first study to prospectively evaluate real-life practices for tuberculosis screening at multiple HIV care clinics.

We found that providers’ initial tuberculosis suspicion rate was only 12.6% in the first audit. We consider this to be low, especially in the context of the high tuberculosis prevalence in Ghana (4) and the high rates of HIV/tuberculosis co-infections observed in resource-limited settings (28). The prevalence of tuberculosis among PLHIV in Ghana is not systematically described, but we have recently reported a culture-confirmed tuberculosis prevalence of 12.7% among PLHIV eligible for ART from a large teaching hospital in Ghana (29). In this study, tuberculosis suspicion rate was highest in consultation with the younger and newly diagnosed HIV-positive patients. Tuberculosis suspicion rate was very low for patients receiving ART (9%) and at follow-up consultations (10%), although PLHIV remain at risk of tuberculosis throughout the course of HIV disease also after initiating ART (30–32).

Our study showed that healthcare providers deviate from recommended guidelines and standards, despite these being evidence-based. Providers did not consistently suspect tuberculosis in patients presenting with cough ≥2 weeks and less so in patients with any of the four symptoms included in the WHO tuberculosis symptom screen. While prolonged cough previously has been identified as the most common symptom screened for in relation to HIV-associated tuberculosis (14), the more broadly defined WHO-TB symptoms seem less well recognised by the healthcare providers as predictive for tuberculosis.

Healthcare providers failed to refer a large proportion of patients suspected of tuberculosis for sputum smear microscopy. Other studies have also recognised low referral rates for tuberculosis diagnostic tests as a ‘leaky’ step in the tuberculosis diagnostic cascade (13, 15). Further down the diagnostic cascade, additional leaky steps have been identified including low rates for completion of the tuberculosis diagnostic tests (33, 34) and high rates of individuals that never start treatment despite a positive test (35). Screening guidelines encourage that any individual with a positive WHO-TB screen, including individuals with cough ≥2 weeks, should be referred for further diagnostic tests regardless of whether the health provider suspects tuberculosis (9). We found very low referral rates for patients with a positive WHO-TB screen (27.5%). This could reflect that the WHO-TB screening algorithm is less well integrated at HIV care sites for intensified tuberculosis case finding. Healthcare providers may be reluctant to overburden the often already challenged tuberculosis laboratories. In our study, more than one-third (34.1%) of the patients presented with a positive WHO-TB screen. In studies of HIV-infected individuals eligible to start ART, up to 90% had a positive WHO-TB screen (36, 37) and the consequence of referring all for sputum microscopy could be dire for a fragile health system. The WHO's TB-screening guideline favours a high negative predictive value to identify those individuals, unlikely to have tuberculosis (i.e. none of the four symptoms reported) where IPT could be started to prevent tuberculosis (7, 9). IPT to PLHIV is not programmatically implemented in Ghana, and this may further contribute to low adherence to the WHO-TB-screening guidelines.

Major progress has been made in developing accurate and rapid diagnostic tools like the Xpert^®^ MTB/RIF (Cepheid, USA). However, implementation of diagnostic tools like the Xpert MTB/RIF has not yet been able to demonstrate reduced mortality in randomised control trials in Africa, despite the great potentials of the test (38, 39). The trials emphasised that the impact of even the most promising tuberculosis diagnostic test is compromised if not coupled to good standards of clinical care (40). The first steps for tuberculosis screening to be effective, regardless of the test, are that healthcare providers suspect tuberculosis in individuals with relevant signs and symptoms and refer them for further diagnostic tests. In our study, inadequate tuberculosis suspicion and low referral rates for sputum microscopy represent low standards of HIV care and a missed opportunity for tuberculosis case detection. We observed that tuberculosis suspicion rates among healthcare providers increased and standards of care improved with audit and feedback. However, we noted a heterogeneous tuberculosis suspicion rate across healthcare providers, and whereas some providers improved performance significantly after feedback, others did not change practice for tuberculosis screening. Furthermore, there was a trend of higher tuberculosis suspicion among nurses than medical doctors, although it levelled out in the second audit. Moreover, tuberculosis suspicion rate was significantly higher in males in the second audit while not in the first audit, and also in age groups, there was a shift in significant differences. Feedback was, in our study, not tailored to specific staff categories or site-specific barriers for tuberculosis screening. It is possible that audit and feedback, as part of a multi-faceted intervention with targeted initiatives, could improve performance further (16). The tool we used for audit and feedback was simple and operational in the daily work of the healthcare providers and effective to identify quality concerns in provider practices. The same tool could be used to monitor and ensure changes in practices after implementation of other interventions to improve tuberculosis case finding. In Ghana, the NTP has recently modified the algorithm for systematic tuberculosis screening of all outpatient attendees. The audit and feedback tool described here is currently being adapted for programmatic implementation within the health system context and challenges to evaluate the revised systematic tuberculosis screening of outpatients.

Our study has limitations. Healthcare providers had to dedicate extra time to fill in the registration form after consultation. In a setting with a high patient flow, this might be difficult and may compromise participation in the audit and quality of entries. Therefore, we instructed the participants to register only the first 15 consultations per day and limited the requirement for details so that entries in the form took <2 minutes. We did not include patient identifiable information and could, therefore, not ascertain if the same patient was entered several times. However, we aimed to characterize the consultation practices rather than the patients. Furthermore, we did not have the capacity to follow up on patients to report the actual test results for patients referred and cannot provide information on the rates for completing a test or starting treatment for tuberculosis given a positive test.

Another limitation is that participants may have registered what they perceived as the correct practice rather than what truly happened. This could have been avoided by direct observation of practices that was out of scope for this study. The design of our study prevents us to draw causal inferences, and other factors than the audit and intervention could be responsible for the change seen in performance. Some of the changes observed could be related to concurrent activities to increase tuberculosis case detection or may reflect the increased focus on tuberculosis screening aroused by the national prevalence survey. In future studies, it could be beneficial to include a control group or a qualitative assessment of contextual factors that could have affected the changes in performance observed in our study.

## Conclusion

A simple audit tool for quality development identified a low tuberculosis suspicion rate and substandard performance of healthcare providers’ tuberculosis screening practices at HIV care clinics in Ghana. Performance improved after audit and feedback to the healthcare providers, in particular for adherence to the WHO guidelines for tuberculosis screening of PLHIV. Flaws in healthcare provider practices for tuberculosis screening and referral for sputum smear microscopy at this level represent a lost opportunity for tuberculosis case detection and inadequate HIV care. To harvest the full benefit of new promising diagnostic technologies, the practices of healthcare providers must come more into focus and effort must be placed on identifying and closing gaps in the quality of clinical care.

## Supplementary Material

Tuberculosis screening in patients with HIV: use of audit and feedback to improve quality of care in Ghana

## Paper context

The success of improved tuberculosis diagnostic test relies on healthcare providers’ practices and adherence to evidence-based tuberculosis screening guidelines. We found low tuberculosis suspicion rates among healthcare providers at HIV care clinics in Ghana compounded by low referral rates for sputum smear microscopy. Adherence to guidelines improved with audit and feedback. Our study proposes a simple method of prospective audit and feedback to quantify substandard performance and improve the quality of clinical practices for tuberculosis screening.
