# Supplementary material for: Tuberculosis screening in patients with HIV: use of audit and feedback to improve quality of care in Ghana
Source: Glob Health Action. 2016 Aug 26;9:10.3402/gha.v9.32390. doi: 10.3402/gha.v9.32390 (PMC5002398; doi:10.3402/gha.v9.32390)
Supplement: Tuberculosis screening in patients with HIV: use of audit and feedback to improve quality of care in Ghana [file GHA-9-32390-s001.pdf]

Supplemental material
To Bjerrum S, Bonsu F, Hansen-Nortey NN et al. ***Tuberculosis screening in patients with HIV: Use of audit and feedback to improve quality of care in Ghana***

A. The Audit Project Odense registration form

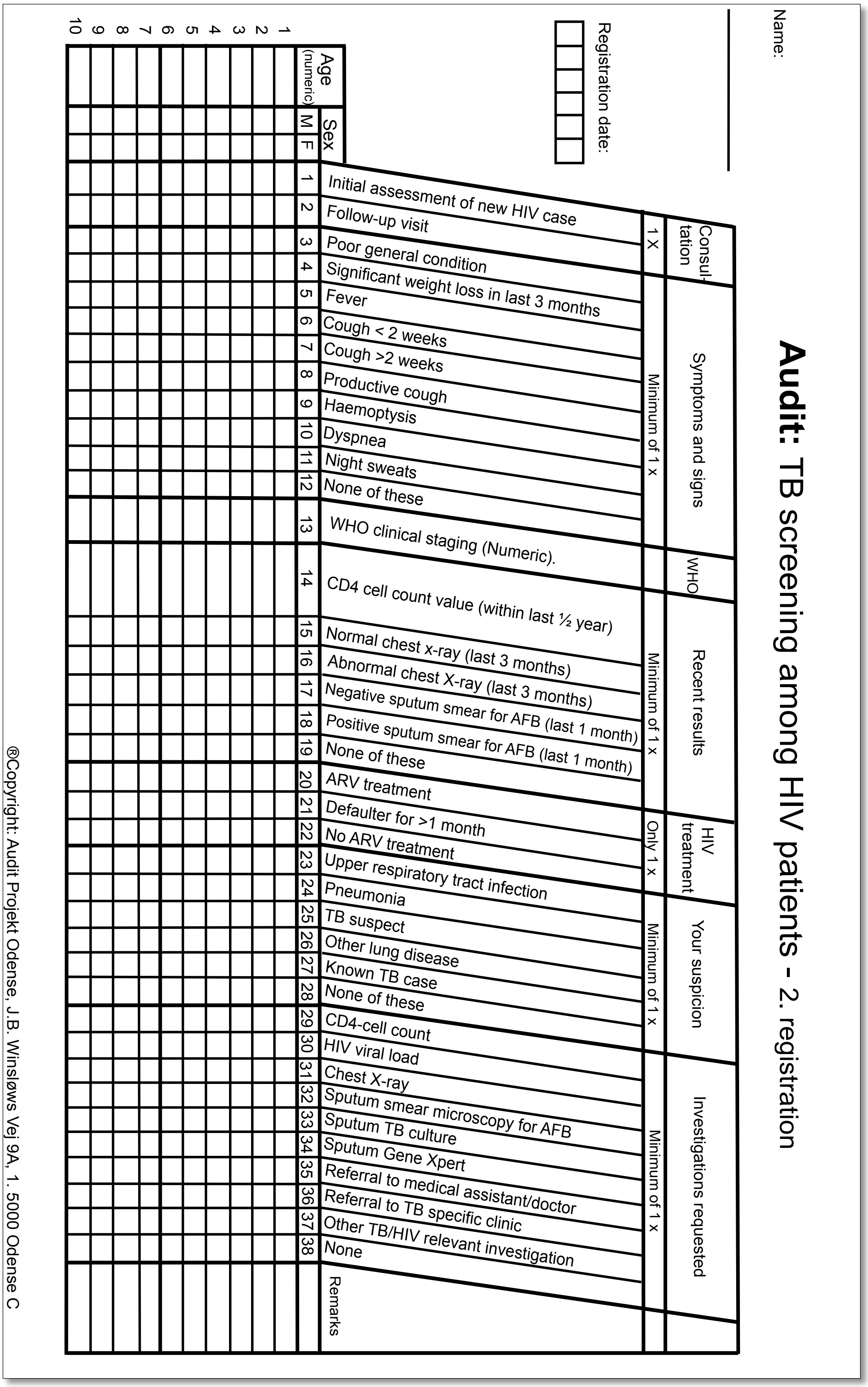

Each form can capture details of ten patient consultations with registration of one patient consultation per row.

Supplemental material
To Bjerrum S, Bonsu F, Hansen-Nortey NN et al. *Tuberculosis screening in patients with HIV: Use of audit and feedback to improve quality of care in Ghana*

B. Summary instructions for the participants to fill-in the registration form

Instructions for the registration chart	
**Registrant**	
Name	Name of registrant (healthcare provider)
Date	Registration date, written as DDMMYY
**Patient consultation**	
Age and Sex	Register age in years<br>For sex register M for male and F for female
Consultation	Tick the consultation according to type; Initial assessment of a new HIV case coming or Follow-up consultation with a known case previously seen in the clinic.
Symptoms and signs	Tick off minimum 1 column<br>Tick the symptoms and signs that patient presented during consultation being self-reported, verified by clinical examination or derived from your best judgement of the patient. If the patient had no complaint or presented with other symptom/signs than those stated in this group remember to tick in "None of these".<br>For "Poor general condition" tick if the patient is cachectic, unable to stand on own feet, not able to communicate or in a similar severe condition.<br>For "Significant weight loss" tick if unintentional weight loss of ≥ 5 % bodyweight within 3 month either self-reported (if new case) or verified by weight noted at previous consultations.<br>For "Fever" tick if self-reported fever or raised temperature measured by thermometer.<br>For "Dyspnoea" tick if self-reported breathlessness and/or patient is dyspnoeic at consultation.
WHO	Indicate the WHO clinical stage in numeric value; 1,2,3,4 *if* a WHO clinical staging is done during the consultation, or *if* you use a previous staging in your evaluation of the patient.
Recent results<br>- Tick off minimum 1 column	Tick if any of the results are present at the consultation.<br>For "CD4 cell count", state the actual value if available within the last 6 months.<br>If none of the results mentioned are available tick in "None of these".
HIV treatment<br>- Only 1 tick	Tick if patient is already on ARV, has been on ARV but defaulted for minimum 1 month or if the patient has not yet started ARV
Outcome<br>- Only 1 tick	Tick your primary diagnosis/suspicion based on the consultation. If there is more than one diagnosis you have to decide which one you think is the most important.
Investigations requested<br>- Tick off minimum 1 column	Tick if you made any of the requests mentioned and/or if you did take any of the actions stated. If none of the actions mentioned was taken tick in "none".
